# ASO Author Reflections: Patient-Reported Outcomes of the CAIRO6 Phase II Trial

**DOI:** 10.1245/s10434-023-13292-y

**Published:** 2023-02-28

**Authors:** Checca Bakkers, Koen P. Rovers, Anouk Rijken, Simon W. Nienhuijs, Ignace H. J. T. de Hingh

**Affiliations:** Department of Surgery, Catharina Cancer Institute, Eindhoven, The Netherlands

## PAST

Cytoreductive surgery (CRS) with hyperthermic intraperitoneal chemotherapy (HIPEC) has been recommended in most (inter)national guidelines for selected patients with colorectal peritoneal metastases (CPM).^1^ While the additional benefit of HIPEC after CRS is currently a topic of discussion, there is also no consensus on the role of perioperative systemic therapy in addition to CRS/HIPEC for resectable CPM. As a result, the administration and timing of perioperative systemic therapy in this patient group is heterogenous worldwide, and insight into its efficacy and burden is lacking.^2^ In the absence of clinical trials investigating this topic, the CAIRO6 trial was commenced in 2017.^3^ This investigator-initiated, parallel-group, open-label, phase II/III, superiority trial randomizes patients with resectable CPM to upfront CRS/HIPEC alone or CRS/HIPEC with perioperative systemic therapy. As part of the phase II trial, patient-reported outcomes (PROs) were compared between both treatment groups to evaluate the burden of perioperative systemic therapy in this setting.^4^

## PRESENT

The present study showed that all predefined PROs were comparable between both groups (i.e., no statistically significant differences) and all returned to baseline at 3 or 6 months postoperatively. Secondary explorative analyses in patients receiving perioperative systemic therapy showed statistically significant and clinically relevant worsening of several PROs (fatigue, hair loss, loss of taste, and loss of appetite) following neoadjuvant treatment. However, all of these (except loss of appetite) returned to baseline scores at 3 or 6 months after CRS/HIPEC.^4^ As this is the first published comparison of PROs in this setting, the present study provides relevant insight into the burden of perioperative systemic therapy for patients with resectable CPM. The previously published CAIRO6 phase II trial report suggested that perioperative systemic therapy in this setting is feasible, well tolerated, and able to induce relevant response of CPM.^5^ Together with the acceptable burden of perioperative systemic therapy, as found in the present study, continuation of the CAIRO6 phase III trial is justified.

## FUTURE

The CAIRO6 phase III trial is currently open for inclusion in eight Dutch expert centers and one Belgian expert center. The primary endpoint is 3-year overall survival. With the hypothesis of a 15% increase in 3-year overall survival favoring patients receiving perioperative systemic therapy (65% vs. 50% in patients undergoing upfront CRS/HIPEC), 358 patients need to be included.^1^ Major secondary endpoints are progression-free survival, disease-free survival, and major postoperative morbidity, and PRO analyses will be performed again in the entire cohort. Given the positive feasibility, safety and tolerability results of phase II, alongside the suggested tumor response in CPM, the CAIRO6 investigators hope to complete inclusion of phase III within the foreseeable time, in order to clarify the role of perioperative systemic therapy in this setting and to standardize and improve the treatment of patients with CPM globally.
